# Replicability of a resting-state functional connectivity study in profound early blindness

**DOI:** 10.3389/fnsys.2025.1547276

**Published:** 2025-04-28

**Authors:** Negin Nadvar, Corinna Bauer, Zahide Pamir, Lotfi B. Merabet, Vincent Koppelmans, James Weiland

**Affiliations:** ^1^Department of Biomedical Engineering, University of Michigan, Ann Arbor, MI, United States; ^2^Laboratory of Neuroimaging and Vision Science, Department of Radiology, Massachusetts General Hospital, Harvard Medical School, Boston, MA, United States; ^3^Laboratory for Visual Neuroplasticity, Department of Ophthalmology, Massachusetts Eye and Ear, Harvard Medical School, Boston, MA, United States; ^4^Department of Psychology, Bilkent University, Ankara, Türkiye; ^5^Department of Neuroscience, Aysel Sabuncu Brain Research Center, Bilkent University, Ankara, Türkiye; ^6^Department of Psychiatry, University of Utah, Salt Lake City, UT, United States; ^7^Huntsman Mental Health Institute, University of Utah, Salt Lake City, UT, United States; ^8^Department of Ophthalmology & Visual Sciences, University of Michigan, Ann Arbor, MI, United States; ^9^Biointerfaces Institute, University of Michigan, Ann Arbor, MI, United States

**Keywords:** ocular blindness, resting-state functional connectivity, fMRI, whole-brain connectivity, replication studies

## Abstract

It has been shown that the choice of preprocessing pipelines to remove contamination from functional magnetic resonance images can significantly impact the results, particularly in resting-state functional connectivity (rsFC) studies. This underscores the critical importance of replication studies with different preprocessing methodologies. In this study, we attempted to reproduce the rsFC results presented in an original study by Bauer et al. in 2017 on a group of sighted control (SC) and early blind (EB) subjects. By using the original dataset, we utilized another widely used software package to investigate how applying different implementations of the original pipeline (RMin model) or a more rigorous and extensive preprocessing stream (RExt model) can alter the whole-brain rsFC results. Our replication study was not able to fully reproduce the findings of the original paper. Overall, RExt shifted the distribution of rsFC values and reduced functional network density more drastically compared with RMin and the original pipeline. Remarkably, the largest rsFC effects appeared to primarily belong to certain connection pairs, irrespective of the pipeline used, likely demonstrating immunity of the larger effects and the true results against suboptimal processing. This may highlight the significance of results verification across different computational streams in pursuit of the true findings.

## 1 Introduction

The emergence of high-dimensional neuroimaging data has given birth to new challenges, with concerns being raised in recent years regarding reproducibility and control for biological and nonbiological sources of noise in MR-derived signal (Klapwijk et al., 2021; Kriegeskorte et al., 2009; Lindquist et al., 2019). While neuroimaging is a useful tool for answering scientific questions, decisions are made at each stage of analysis to mitigate sources of potential noise, including data collection, choice of spatial and temporal image processing techniques, statistical assessment, and order of their application, which can each impact the study outcomes. Within the past decade, numerous algorithms have been developed to minimize the effect of contamination of this data from noise and artifacts and best practices have been proposed to optimize data collection and analysis (Maumet et al., 2016; Nichols et al., 2017; Poldrack et al., 2008, 2017), although there has yet to be consensus in the field.

Functional magnetic resonance imaging (fMRI) studies, in particular, are vulnerable to spurious signals and challenges in replicating results. One investigation exposed a large number of studies with inflated correlation results between the fMRI blood-oxygen-level-dependent (BOLD) response and behavioral measures (Vul et al., 2009). The questionably large correlations in these studies were attributed to the biased methods adopted for correlation analysis. Statisticians also probed into some of the widely used fMRI analysis packages and discovered that some of these methods in effect inflate the rate of false positives (Eklund et al., 2016). FMRI studies are also often underpowered (Marek et al., 2022), potentially leading to larger type I error and lower positive predictive values (Button et al., 2013). These are a few examples of how spurious results in fMRI studies can be partially attributed to inappropriate statistical methodologies and/or inherently low statistical power.

Additional factors rendering fMRI studies prone to poor reproducibility are the flexibility in selection and combination of analysis techniques and insufficient reporting of detailed procedures in design, acquisition, and computation (Poldrack et al., 2008). Earlier modeling studies indicated that greater flexibility in design and computational approaches is associated with a greater probability of false positive errors (Ioannidis, 2005). This is true of fMRI in which nearly each study may have a unique analysis pipeline (Carp, 2012a), resulting in significant variability in estimates of the strength, location, and statistical significance of activations (Carp, 2012b). With the emergence of open-source neuroimaging analysis tools, additional sources of analytical variability have recently been acknowledged, as analysis pipelines for the same overarching procedure are implemented differently in nearly each software package (Poldrack et al., 2017). Consequently, replicating fMRI studies becomes challenging, as algorithm implementation and the ordering of preprocessing steps have been shown to influence study results (Carp, 2013; Lindquist et al., 2019). As a result, replication studies using a different dataset, computational approach, or pipeline are critically important in fMRI studies to validate the previously published findings or to reveal sources of variability.

In the present replication study, we attempted to reproduce the resting-state functional connectivity (rsFC) findings of an original study by Bauer et al. (2017). The original work explored the alterations in rsFC in a group of subjects with early ocular blindness compared with sighted controls. The study reported increased temporal correlations in BOLD signal derived from rsFC between inferior frontal and temporal areas, as well as a more predominant decrease in correlations between occipital and frontal, occipital and somatosensory/motor, temporal and parietal, temporal, and frontal cortices and within the temporal in early blind as compared to the sighted control. Importantly, some of the works prior to this primary study reported decrease in visual-somatosensory and visual–auditory rsFC (Bedny et al., 2011; Burton et al., 2014; Striem-Amit et al., 2015) and increased rsFC in visual-frontal and visual-parietal areas following blindness (Burton et al., 2014; Heine et al., 2015; Watkins et al., 2012). In our present replication study, by using the dataset utilized for the original work, we aimed to repeat the analysis with a different, but widely used software package for the analysis of rsFC and to include more rigorous handling of the effects of known noise and artifacts. The aims of this study are (1) to try replication by using the same preprocessing steps as the original study but with different implemented algorithms, and (2) to evaluate replicability when using a more extensive pipeline than the original study. In our first replication approach, we cleaned the data by using the minimal preprocessing procedure similar to the original paper. In the second attempt, we incorporated some of the main preprocessing and denoising protocols typically suggested to reliably prepare the rsfMRI data for rsFC analysis.

## 2 Materials and methods

### 2.1 The target of replication

In this replication study, we utilized neuroimaging data acquired as a part of the original research article (Bauer et al., 2017). Briefly, 9 sighted control (SC) and 11 early ocular blind (EB) subjects were included in the analysis of rsFC. Anatomical T1W scans (TE = 3.1 ms, TR = 6.8 ms, flip angle = 9°, voxel size 0.98 × 0.98 × 1.20 mm, turbo spin echo) and one 7 min rsfMRI run (TE = 30 ms, TR = 3,000 ms, flip angle = 80°, voxel size 2.75 × 2.75 × 3.00 mm, single-shot EPI) were obtained on a 3 T Philips Intera Achieva scanner. Subjects were blindfolded and were not guided to perform any directed task within the scanner. For each subject, a static fieldmap acquisition was also completed (TE1 = 2.3 ms, TE2 = 4.6 ms, TR = 20 ms, flip angle = 10°, voxel size 1.02 × 1.02 × 3.00 mm, fast field echo). The experiment was approved by the Institutional Review Board at the Massachusetts Eye and Ear Infirmary, Boston, MA, United States, and a written consent form was obtained from each participant.

By keeping the data identical, we focused on replicating the resting-state functional connectivity results, presented in the original paper, using a different set of methodologies. Therefore, this work sought to reveal whether the same results are obtained by altering the analysis protocol. This replication work also helped us identify a coding error in implementing the correction for multiple comparisons in the original study. Specifically, we identified an unintentional computational error in the false discovery rate (FDR) calculation used in the multiple comparison corrected contrast results presented in Figure 3B and Table 4 in the original paper. After applying an updated FDR correction to both the replication results and the preprocessed data from the original paper, it was found that none of the originally reported connections from the contrasts of interest survived the multiple comparison correction. Undeniably, the discovery of null scientific results and reporting them remains crucial and valuable to the advancement of science and thus is just as important as reporting the significant results. In addition, it is helpful to communicate the uncorrected results, especially to reveal any underlying trends in the data (Poline et al., 2006; Poldrack et al., 2008). Consequently, we aimed to focus on reproducing the initial uncorrected results presented by the primary research article and took a closer look at the influence of each pipeline on the effect sizes of interest.

### 2.2 Differences and similarities between the pipelines under study

The original paper reports that analysis was mainly implemented by the publicly available software package FSL FEAT v. 6.00 (Jenkinson et al., 2012) and FreeSurfer v. 5.3.0 (Fischl, 2012) to perform skull-stripping using BET, static fieldmap correction, realignment and temporal regression of motion parameters and their derivatives. The rsfMRI data was coregistered to T1W structural scans using boundary-based registration (BBR) (Greve and Fischl, 2009) and 68 parcellations from the Desikan-Killiany atlas (Desikan et al., 2006) were reverse transformed into subject-specific space to serve as regions of interest (ROIs) using FreeSurfer. A custom MATLAB (Mathworks, Natick, MA, United States) script was used to bandpass-filter the functional data between 0.01 Hz and 0.1 Hz, detrend the BOLD signal, calculate Fisher transformed Pearson correlation coefficients (z) between each ROI pair and create a 68×68 functional connectivity matrix for each subject. Group-level results were created by averaging the z values for each connection within each group. Between-group contrast maps were computed by applying a two-sample *t*-test to z values of both groups.

We devised two different preprocessing pipelines using the CONN toolbox 20.b (Whitfield-Gabrieli and Nieto-Castanon, 2012) to replicate the original results, which were generated using FSL v. 6.00 (Jenkinson et al., 2012). In the first pipeline, we attempted to perform steps as similar as possible to the primary paper. We call this the replication minimum (RMin) model. In the second pipeline, we incorporated a more comprehensive set of processing steps, strongly recommended for rsfMRI data (Andersson et al., 2001; Molloy et al., 2014; Muschelli et al., 2014), for the removal of unwanted contamination with noise and artifacts prior to the subsequent statistical analysis. We refer to this scheme as the extensive replication (RExt) model. The original work and the replication models performed the preprocessing and ROI extraction in subject-specific space to minimize the effect of between-subject variability (Bijsterbosch et al., 2020).

Similar to the original article, the RMin model implemented a subject-specific volumetric analysis, used Desikan-Killiany atlas to parcellate the brain, and incorporated static fieldmap correction, realignment, temporal linear regression of motion parameters, band-pass filtering, and detrending. However, as the Table 1 shows, although the pipeline steps are similar, there are inherent differences stemming from different algorithms used in FSL and CONN to implement each step. Specifically, the B0 unwarping used in the original study is based on a method that applies both realignment and susceptibility distortion correction at the same time to reduce effect of interpolation on blurring (Jenkinson et al., 2002; Jenkinson and Smith, 2001). In the RMin model, the CONN toolbox uses SPM analysis package that in turn relies on the algorithm implemented in the Fieldmap toolbox to correct the geometric distortion of the EPI images (Jenkinson, 2003; Jezzard and Balaban, 1995). Additionally, the head motion is corrected by applying a rigid body transformation, but while the original study used MCFLIRT (Jenkinson et al., 2002), the RMin model used a procedure described in Friston et al. (1995). Coregistration of low-resolution functional images to high resolution structural images in the original work uses the BBR method in FreeSurfer, however the RMin model uses a modified version of a prior work by Collignon et al. (1995) with a change in the interpolation technique for a smoother cost function and faster convergence.

**Table 1:** Side-by-side comparison of the 3 pipelines under study.

	Original paper	RMin model	RExt model
Analysis tool	FSL FEAT 6.00 and FreeSurfer 5.3.0	CONN 20.b/SPM12 and FreeSurfer 7.1.1	CONN 20.b/SPM12 and FreeSurfer 7.1.1
Preprocessing space	Subject-specific volumetric	Subject-specific volumetric	Subject-specific Surface-based
Atlas	Desikan Killiany	Desikan Killiany	Desikan Killiany
Static fieldmap correction	Yes (Jenkinson and Smith, 2001; Jenkinson et al., 2002)	Yes (Jezzard and Balaban, 1995; Jenkinson, 2003)	Yes (Andersson et al., 2001)
Fieldmap-by-motion interaction correction	No	No	
Realignment	Yes (Jenkinson et al., 2002)	Yes (Friston et al., 1995)	
Slice-timing correction	No	No	Yes
Coregistration to subject-specific volumetric space	Boundary-based registration (Greve and Fischl, 2009)	Rigid body transformation (Collignon et al., 1995)	Rigid body transformation (Collignon et al., 1995)
Resampling to subject-specific surface space	N/A	N/A	Yes
Smoothing	No	No	Yes
Motion parameters regression	Yes	Yes	Yes
aCompCor	No	No	Yes
Scrubbing	No	No	Yes
Effect of (rest) session	No	No	Yes
Band-pass filtering	Yes (0.01–0.1 Hz)	Yes (0.01–0.1 Hz)	Yes (0.01–0.1 Hz)
	DCT (performed by CONN)	DCT	DCT
Temporal filtering done after regression	Yes	Yes	Yes
Detrending	Yes	Yes	Yes

Both (Min and Ext) Replication Models used CONN toolbox vs. FSL toolbox in the original paper. In the RMin model, the goal was to maintain the same preprocessing steps as the original paper, although the implementation of these steps was different between the 2 toolboxes. The RExt model attempted to correct for the influence of additional noise and artifact sources known to be crucial in reducing the false positive rate in rsFC analysis. Differences between each replication model and original paper are highlighted in gray. aCompCor, anatomical component-based noise correction; DCT, discrete cosine transform.

In the RExt model, preprocessing was performed in subject-specific surface space using the same atlas, range, and method for bandpass filtering and detrending as the original paper. However, in this model, we supplemented the preprocessing steps originally included in the primary study with additional protocols to remove the residual spatial and temporal distortions still present in the data. Importantly, the RExt model additionally corrected for the susceptibility distortion-by-motion interaction. RExt model combined the static fieldmap correction with realignment and susceptibility distortion-by-motion interaction correction into a single step in CONN, which uses SPM12 Realign & Unwarp procedure (Andersson et al., 2001). The susceptibility distortion-by-motion interaction is the degree to which motion interacts with deformation field and is approximated by the derivative of deformation field in relation to motion, which estimates the position-dependent distortions. Using B-spline interpolation, the functional image is then unwarped and resampled to match the reference image’s deformation field. Neither the original paper nor the RMin model incorporated correction for this interaction term. Additionally, the RExt model corrected for the temporal offset in slice acquisition times (Henson et al., 1999). Scrubbing was also performed in CONN to identify potential outlier scans and temporally regress them out. A scan was considered as outlier whenever the global BOLD signal changed more than 5 standard deviations or when a composite motion measure exceeded 0.9 mm. To calculate this motion measure, CONN assumes a 140 × 180 × 115 mm bounding box around the brain and the composite motion measure is computed as the largest displacement among six points each being placed at the center of 6 faces of the bounding box. The rsfMRI data was then resampled at the location of surface projections estimated for each subject using FreeSurfer. In order to increase the signal-to-noise ratio, surface-based smoothing with 5 mm FWHM was further applied (Hagler et al., 2006). Also, since no physiological data was recorded during the acquisition, in the RExt model, we decided to utilize the anatomical component-based noise correction (aCompCor) procedure (Behzadi et al., 2007). In this method, noise is modeled from the white matter and cerebrospinal fluid areas, as the average BOLD signal as well as the Principal Component Analysis first 4 components of the BOLD signal in these areas. These noise components were temporally removed using a general linear model, along with the previously extracted motion parameters and scrubbing confounds. Finally, the resting session block was convolved with the hemodynamic response function and modeled as another source of noise to account for the initial magnetization transients.

### 2.3 Replicated and novel analyses performed

Similar to the original paper, for the replication streams, Pearson correlation coefficients between ROI pairs were calculated and Fisher z-transformed to ensure a normal distribution. Each subject’s Fisher z values were then used to create between-group rsFC contrast matrices by applying two-sample *t*-tests to z values across the 2 groups for each connection pair. The resulting *p*-values of the contrasts were thresholded for *p* < 0.05 to yield uncorrected results for the rsFC matrices. Since connections are bidirectional, less than half of the connection pairs (68*67/2 = 2,278) were redundant in each rsFC matrix. As a result, we merged the positive (EB > SC) and negative (EB < SC) matrices together in a single rsFC map that contained both contrasts. This combined rsFC matrix was created for results of both replication models and is a reproduction of Figure 3A in the original paper. In addition, to facilitate the comparison between the replication and the original work, we calculated density of each of the resulting connectivity matrices by finding the percentage of connections that survived the thresholding (*p* < 0.05) for each pipeline and contrast. The number of connections in the replication work that survived this threshold and were common with the results from the original work were also calculated as the overlap with the original (OWO) results for each replication pipeline and contrast. Similarly, the percentage of such overlap (OWO%) was also computed as the percentage of thresholded results in the original work that are commonly discovered in the corresponding replication results. The associated formula is indicated in Equation 1, where N_orig_ represents the number of connection pairs in the rsFC matrix that survived the *p* < 0.05 threshold in the original model, OWO represents the number of connection pairs that survived the *p* < 0.05 threshold in both the original and a replication model (RFMin or RExt). The calculations of network density and overlap were not a part of the original article.


(1) $$OWO\%=100\times \frac{OWO}{N_{\mathrm{Orig}}}$$

In addition to replicating the mentioned figures in the original paper, we inspected the distribution of each group’s effect size (z values) that further illustrated the effect of each of the 3 pipelines. We also expanded upon the original analysis to exam network density, which was calculated for each group and each pipeline. Network density was computed in two ways: by finding the percentage of connections that either passed the uncorrected thresholding (*p* < 0.05) or the multiple comparison FDR correction, out of all the possible connections in the network. Distributions of the effects were also compared between each replication model and the original results, using paired samples *t*-test within each study group. Finally, for each group and each analysis stream, the top 1% of all the z values for each pipeline and subject group were extracted and displayed in the corresponding circular connectograms. Overlap of the top 1% of the z values in the replication works with the original result was also computed and reported.

## 3 Results

### 3.1 Replication of between-group contrast results

**Figure 1:**
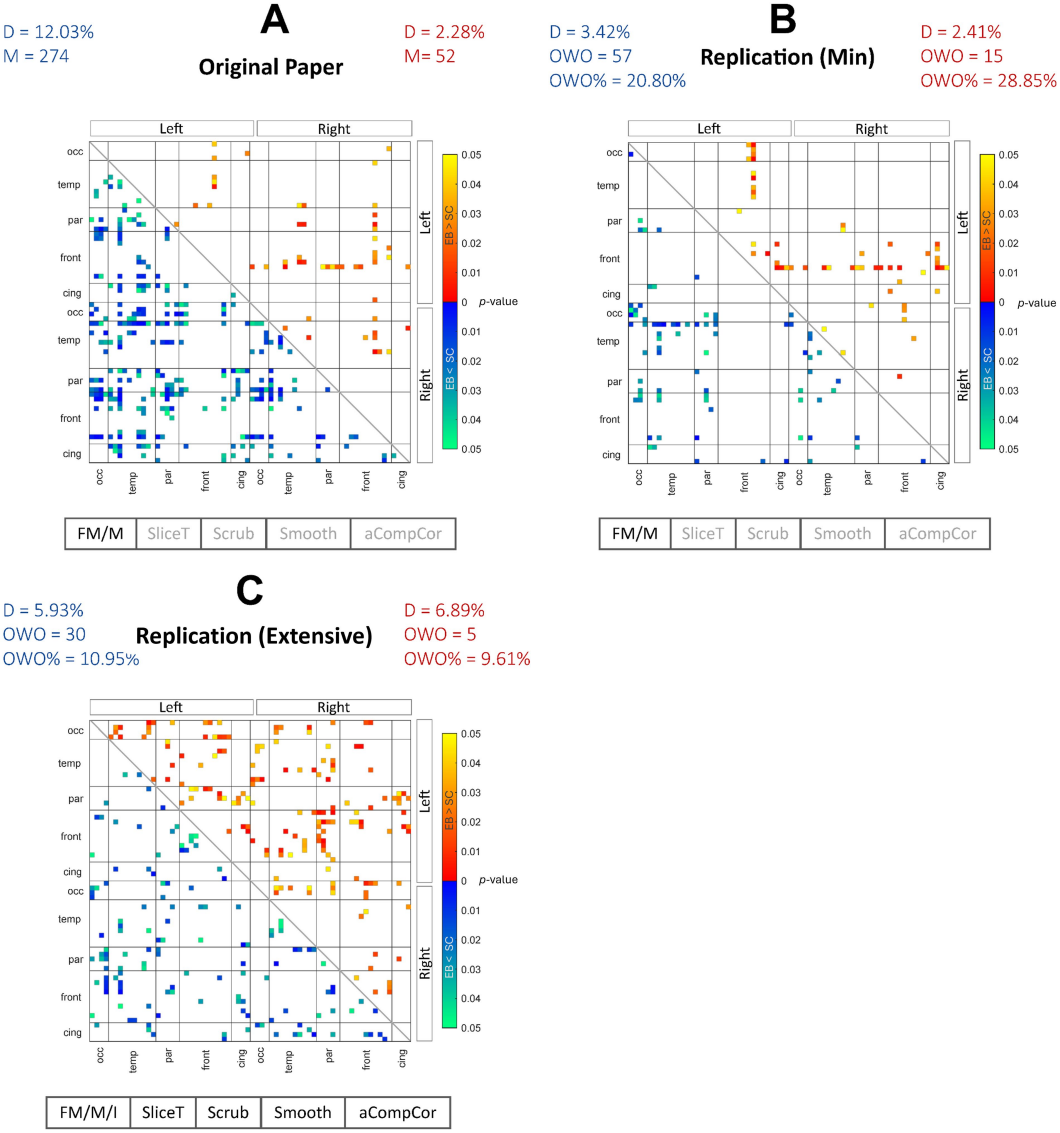
Between-group contrast results for the 3 preprocessing pipelines. Between-group EB > SC (warmer colors) and EB < SC (colder colors) FC contrast results are shown for the original paper **(A)**, the replication min model **(B)** and replication extensive model **(C)**. The EB > SC network density remained relatively unchanged for the replication min model while increased in the replication extensive model compared with the original work. The EB < SC network density decreased in both replication models. Overall, the uncorrected FC network in the replication extensive model showed less overlap with the results from the original work compared with the replication min model. FM, fieldmap correction; M, motion correction; I, fieldmap-motion interaction; SliceT, slice-timing correction; Scrub, scrubbing; Smooth, smoothing; aCompCor, anatomical component-based correction; D, density; M, number of uncorrected pairs in the original work; OWO, overlap with the original; %OWO, percentage of overlap with the original.

We initially aimed to recreate the connectivity matrices for the positive (EB > SC) and negative (EB < SC) contrasts as had been presented in Figure 3A of the original paper. The rsFC matrices containing uncorrected *p*-values (*p* < 0.05) from the original and replication pipelines are indicated in Figures 1A–C, where the warmer and colder colors represent EB > SC and EB < SC contrasts, respectively. The main preprocessing steps incorporated in each pipeline are also recorded below each matrix. Both the RMin and RExt models present different results compared with the original paper. Although, the RMin model produced connection pairs that were shared with the results of the original work, mainly involving the frontal (for EB > SC) and temporal areas (for EB < SC). The density of each network and the (percentage of) overlap with the results from the original work were also computed for each contrast. A qualitative assessment of the contrasts revealed that the density of the positive contrast results was relatively unchanged in the RMin model compared to the original results (2.41% vs. 2.28%) with uncorrected results appearing mainly in the frontal lobe in both cases. On the other hand, the density of the negative contrast results declined in the RMin model vs. the original results (3.42% vs. 12.03%). The results of the RExt model in Figure 1C showed an increase in the network density for the positive contrast (6.89% vs. 2.28%) and a decrease for the negative contrast (5.93% vs. 12.03%). Overall, the RMin model presented a higher overlap of both positive and negative contrasts with the original results (28.35 and 20.80% respectively) compared with the RExt model (9.61 and 10.95% respectively). As explained in Section 2.1, the FDR correction presented in Figure 3B of the original work was determined to be invalid and as a result was not considered for replication in this study. In addition to this qualitative evaluation, formal testing was used in Section 3.2 to determine the statistical significance of the effects of the preprocessing pipeline on individual groups.

**Figure 2:**
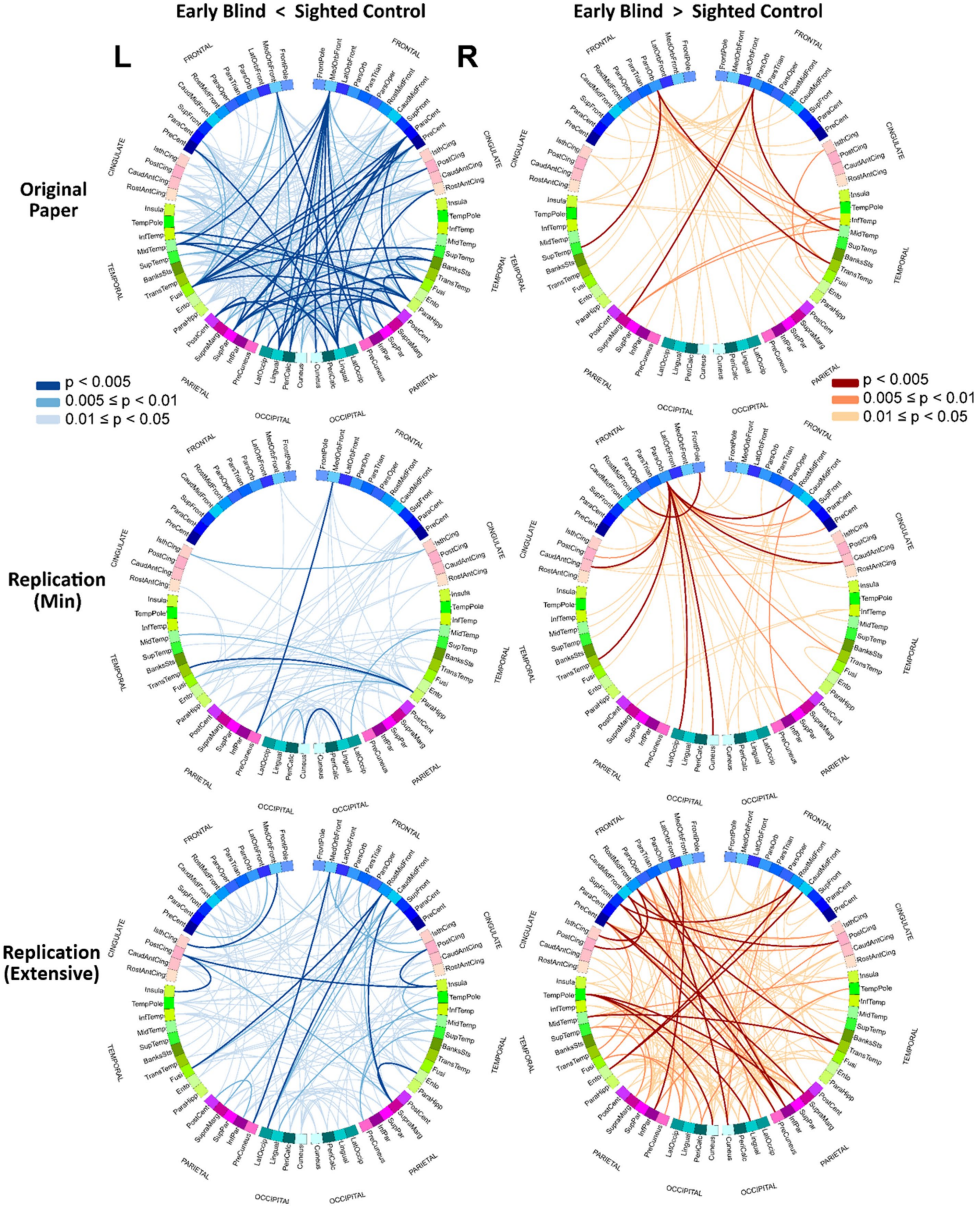
Circular connectograms of uncorrected connections. Circular connectograms of the uncorrected connections (*p* < 0.05) further illustrates the difference between the pipelines’ outcomes. Each connectogram is composed of 68 ROIs extracted from Desikan Killiany atlas with ROIs from left and right brains hemispheres represented on left and right separately. See the Supplementary materials for ROI names abbreviations.

Figure 2 shows the uncorrected (*p* < 0.05) results, for the 2 contrasts in the form of circular connectograms for each of the 3 pipelines under study. Each connectogram incorporates 68 ROIs extracted from the Desikan Killiany atlas, comprising frontal, cingulate, temporal, parietal, and occipital lobes. The left and right hemispheres of the graph represent ROIs in the left and right hemispheres of the brain. The connections between ROIs are color-coded depending on the magnitude of *p*-values for a more granular representation of the uncorrected results. This representation was created to provide a direct comparison with Figures 5C,D in the original paper. A description of the ROI acronyms can be found in the Supplementary Table 1. Although exploring the effect of each preprocessing step was outside of the scope of this study, as an exploratory work, in the Supplementary material (Section 2), we also included the resulting rsFC matrices for alternative pipelines in which some preprocessing steps were removed.

### 3.2 Effect of pipelines on individual groups

**Figure 3:**
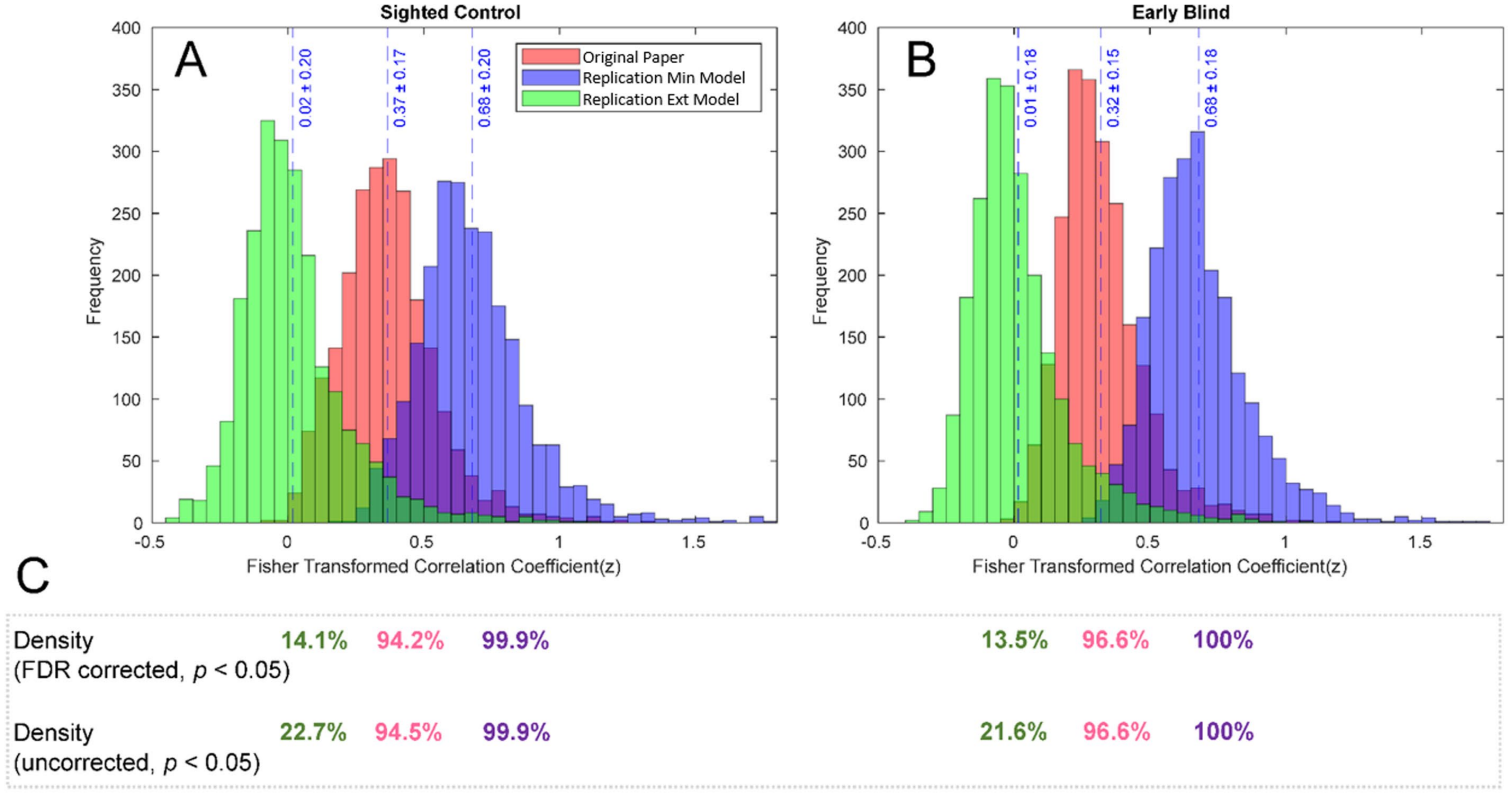
Distribution of FC effects for individual groups. Distribution of FC effects (z values) is shown for SC **(A)** and EB **(B)** groups. While the results of the original study and the replication min model are centered around more positive z values, the replication extensive model shifted the distribution more towards zero. The density of the (corrected and uncorrected) network shown in **(C)** was affected by the selected preprocessing pipeline, with the original work and replication min model presenting density closer to 100% and the replication extensive model presenting a drastically reduced density.

To further analyze the effect of the three different analysis streams, we inspected how each of the approaches influenced the distribution of the effects (z values) as shown in Figures 3A,B for the early blind and control study groups. For both groups, the z values resulted from the original work (shown in red) and the RMin model (shown in violet) are centered around more positive z values (0.37 and 0.68 for SC and 0.32 and 0.68 for EB, for the original paper and RMin model respectively), compared to RExt model where z values are centered closer to zero for both groups (0.02 for SC and 0.01 for EB).

As opposed to the between-group contrast results for rsFC analysis, the individual group results yielded connections that passed the multiple comparison correction. The density of the FC networks after thresholding (uncorrected, *p* < 0.05) and after correction for multiple comparisons (FDR, *p* < 0.05) is summarized for each pipeline and group in Figure 3C. Density of the FC network followed the same trend as the z-distributions, with the RExt model presenting a drastically reduced network density for both corrected and uncorrected results (14.1% and 22.7 in SC, 13.5 and 21.6% in EB), compared with the network density of corrected and uncorrected results from the original work (94.2 and 94.5% for SC, 96.6 and 96.6% for EB) and the RMin model (99.9% for SC and 100% for EB in both corrected and uncorrected results).

In order to statistically evaluate the impact of preprocessing pipelines on the effects, for each group, the distribution of functional connectivity effect values from the original paper was compared with that of RMin and RExt models, using paired samples *t*-tests. This resulted in four tests across the two study groups with all the tests yielding statistically significant differences (*p* < 0.05). The statistics associated with each of these additional tests are summarized in Table 2.

**Table 2:** Summary of statistics of paired samples *T*-tests for comparing functional connectivity effects of the original study with the RMin and RExt models.

	*p*-value	T-statistic	Degrees of freedom	95% CI (of pairs differences)	SD (of pairs differences)
*Original vs RMin (EB)*	<0.001	−74.86	2,277	[−0.37 – 0.35]	0.23
*Original vs RExt (EB)*	<0.001	65.52	2,277	[0.29 – 0.31]	0.22
*Original vs RMin (SC)*	<0.001	−57.38	2,277	[−0.32 – 0.30]	0.26
*Original vs RExt (SC)*	<0.001	68.23	2,277	[0.34 – 0.36]	0.24

CI, confidence interval; SD, standard deviation.

### 3.3 Connection pairs with the largest effects

**Figure 4:**
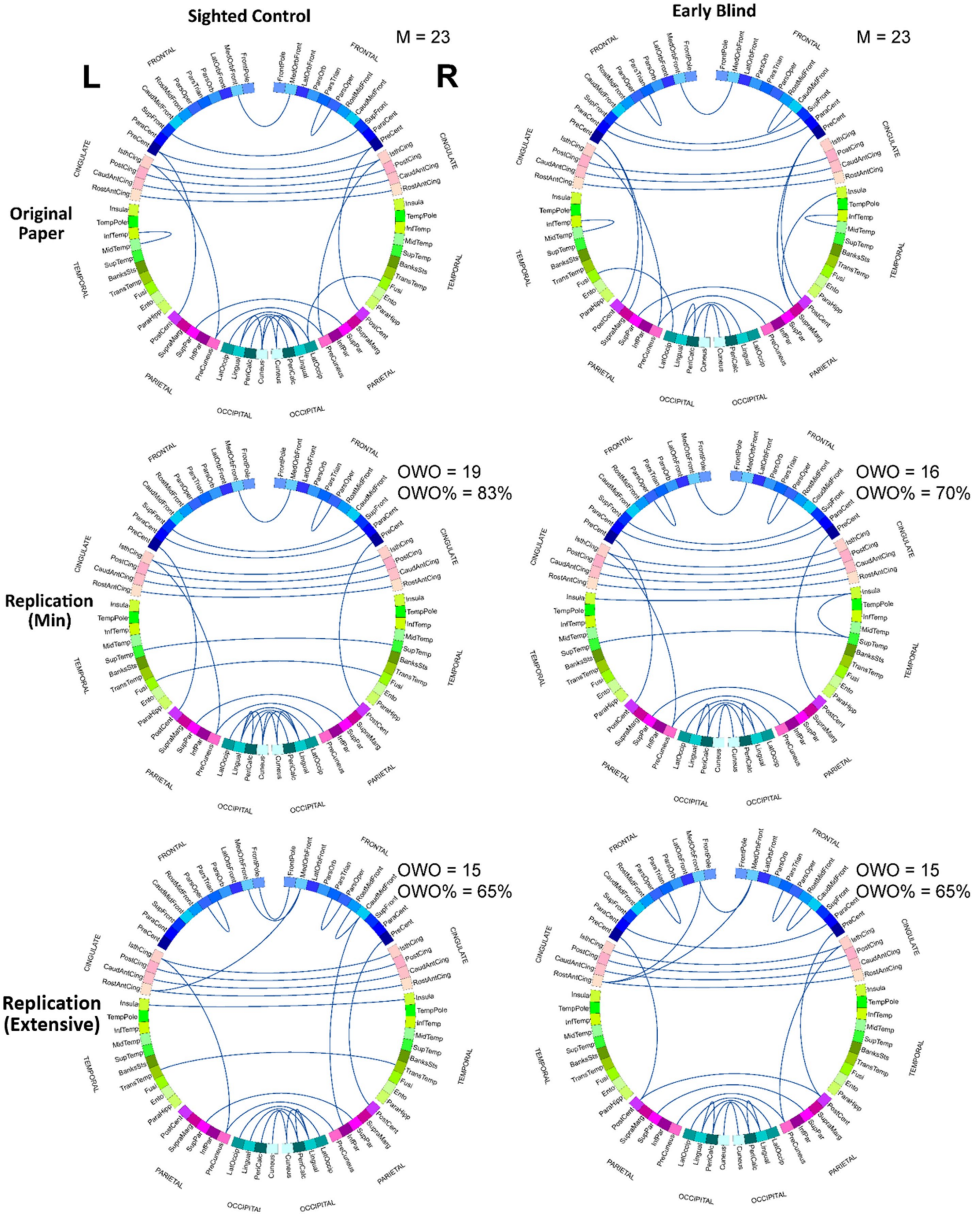
Connection pairs with the largest effects for each pipeline. Connection pairs that constitute the top 1% of z values in the z-distribution (99% percentile and higher) were identified for each pipeline and each group. These pairs are shown as links in the circular connectograms. Both RMin and RExt models present results with notable overlap with the results from the original analysis. M, number of pairs identified in the original study comprising the top 1% of z values; OWO, overlap with the original (number of links in the replication models that overlap with the original results). See the Supplementary material for ROI names abbreviations.

Finally, to uncover the specific connection pairs that more strongly survive each of the three preprocessing pipelines, we identified connections that constitute the top 1% of z values for each group and each pipeline. The results are summarized in the form of circular connectograms in Figure 4. To measure the degree to which the result of each replication stream overlaps with the original pipeline, we calculated the overlap with the original as the number of common connections in the top 1% z values between the original and each replication model. Out of the 23 connections (top 1% of all the z values) in the original work, 19 (83%) and 16 (70%) connection pairs overlapped with the top 1% z values in the RMin model for SC and EB groups, respectively. There were 15 (65%) overlapping connections between the original and RExt models for either SC or EB group. Table 3 indicates the connection pairs that composed the top 1% of effects and were shared among all the 3 pipelines for SC and EB groups. This included both intra- and inter-hemispheric links. As an exploratory work, connectivity pairs comprising the top 1% of the *t*-values for the between-group contrast were also extracted for each pipeline and group. Further details are included in the Supplementary material (Section 3).

**Table 3:** Connections with the largest effects independent of the choice of pipeline.

Sighted control				
**Inter-hemispheric**	**Frontal**	**Cingulate**	**Parietal**	**Occipital**
	MedOrbFront	IsthCing PostCing CaudAntCing RostAntCing	SupPar PreCuneus	LatOccip Cuneus PeriCalc Lingual
**Left intra-hemispheric**	**Cingulate-parietal**			
	IsthCing-PreCuneus			
**Right intra-hemispheric**	**Frontal–parietal**	**Frontal–frontal**	**Occipital-occipital**	
	PreCent-PostCent	ParsTrain-ParsOperc	Lingual-Cuneus	

Early blind				
**Inter-hemispheric**	**Frontal**	**Cingulate**	**Parietal**	**Occipital**
	MedOrbFront and ParaCent	PostCing CaudAntCing RostAntCing	SupPar PreCuneus	Cuneus PeriCalc Lingual
**Left intra-hemispheric**	**Frontal–parietal**	**Frontal–frontal**	**Occipital-occipital**	
	PreCent-PostCent	ParsTrian-ParsOper	Lingual-PeriCalc	
**Right intra-hemispheric**	**Frontal–parietal**			
	PreCent-PostCent			

The table contains the connection pairs that comprised the top 1% of FC effects and were common between all the 3 pipelines for sighted control and early blind groups separately. These links connected the left and right hemispheres (inter-hemispheric) or different ROIs within each left/right hemisphere (intra-hemispheric). CaudAntCing, Caudal Anterior Cingulate; IsthCing, Isthmus Cingulate; LatOccip, Lateral Occipital; MedOrbFront, Medial Orbitofrontal; PostCing, Posterior Cingulate; PreCuneus, Precuneus; PeriCalc, Pericalcarine; PreCent, precentral; PostCent, Postcentral; ParsTrain, Pars Triangularis; ParsOperc, Pars Opercularis; ParaCent, Paracentral; RostAntCing, Rostral Anterior Cingulate; SupPar, Superior Parietal.

## 4 Discussion

The present replication study was not able to reproduce the results of the original paper, using either of the two pipelines used for the replication. Although the between-group contrast FC maps for the RMin model presented connection pairs implicating frontal (for EB > SC) and temporal (EB < SC) regions similar to the findings in the original work. Overall, qualitative comparison showed that the overlap with the original results was less in the RExt model for both contrasts compared with the RMin model.

Both replication models showed a statistically significant difference in the distribution of effect sizes compared with the original study. In other words, neither of the replication models was able to compellingly replicate the effects distributions. A closer examination of the distribution of FC effects after using each pipeline revealed that z values for both the original and RMin models were centered around more positive values whereas the RExt model shifted the effect values towards zero. This, in effect, resulted in the uncorrected and multiple comparison corrected networks that were noticeably less dense in the RExt model compared to the other pipelines under study. In other words, the RExt model reduced the number of positive (and likely false positive) findings. This is due to the expected influence of all the additional processes incorporated in the RExt model; for instance, aCompCor estimates and removes the residual effect of non-neural noise sources like cardiac signal, respiration, and head motion (Behzadi et al., 2007; Muschelli et al., 2014) and has been shown to likely increase the sensitivity and specificity of brain network’s positive rsFC correlations (Chai et al., 2012). Slice timing correction, another step that is excluded from the original study, has been shown to improve the extraction of functional networks and increase the accuracy of the BOLD time series associated with each voxel by removing temporal offsets (Parker and Razlighi, 2019). Additionally, since the structural artifacts, such as motion, can interact with the magnetic field (Bijsterbosch et al., 2020), in the RExt model we incorporated the removal of susceptibility distortion-by-motion interaction. This step provides a better estimate of the fieldmap and its derivative with respect to motion and thus further reduces the residual motion-related variance (Andersson et al., 2001). Finally, the RExt model also included spatial smoothing of the functional data, a well-documented procedure to increase the temporal signal-to-noise-ratio (Brodoehl et al., 2020; Molloy et al., 2014; Triantafyllou et al., 2006). Nevertheless, spatial smoothing is a debatable topic for brain functional networks and the choice of the smoothing kernel can impact the FC networks (Alakörkkö et al., 2017; Triana et al., 2020).

In our RMin model, when we attempted to implement the same preprocessing steps as the original work, both the between-group contrasts and the group-level results still showed discrepancies from the original study. Further investigation into the potential sources of this incongruity revealed that the similar steps are in effect implemented based on different algorithms in the two software packages used. FSL, used in the original work, implements the static field map correction and realignment based on the methods proposed by Jenkinson et al. (2002) and Jenkinson and Smith (2001) and (boundary-based) registration based on Greve and Fischl (2009). In contrast, CONN/SPM 12, used in the RMin model, implements each of these steps with other distinct algorithms (Collignon et al., 1995; Friston et al., 1995; Jenkinson, 2003; Jezzard and Balaban, 1995). Therefore, the incongruity observed between the original study and RMin model may be due to dissimilar implementations of the same steps in the two software packages, a source of irreproducibility previously highlighted in other studies (Carp, 2012b).

Besides the contribution of diverse computational schemes, another factor that can lie at the heart of the irreproducibility of the results from the original study is the low statistical power. It is reasonable that finding study participants with the specific visual deficit under study in the original paper can be complicated, nevertheless the small sample size will result in low power. Collecting rsfMRI data for longer periods from each subject could have minimized this effect (Poldrack et al., 2017).

Interestingly, the highest FC effects (z values) appeared to predominantly belong to specific connection pairs regardless of the analysis pipeline. This demonstrates that large FC effect sizes are more likely to remain immune to the adverse effects of suboptimal preprocessing pipelines. On the other hand, these large z values likely represent the true results, as it has been previously demonstrated that the true positive findings are inclined to replicate more across studies, while this does not hold true for the false positive results (Moonesinghe et al., 2007). The observed overlap in connections among different preprocessing streams in this study may underscore the importance of verifying results across different pipelines in search of the true results; an ultimate goal particularly critical for studying clinical populations.

It is important to emphasize that the findings of this replication study are limited by the specific computational toolboxes used, CONN and SPM, and their release versions. However, the use of a widely used toolbox like CONN enabled us to utilize its comprehensive pipelines that preclude the need for using various application tools for preprocessing pieces. In the RMin model, this allowed us to investigate the impact of different algorithms used for the implementation of the same steps compared with the original pipeline, which was one of the aims of the study. Admittedly, future works are encouraged to also investigate the impact of different implementations of the same computational algorithms for identical preprocessing steps. In addition, the small number of participants and the inherently low statistical power of the study may hinder any reproducibility effort, regardless of the analytical pipeline used. Lastly, although the three processing streams under study were conceptually and qualitatively compared throughout the study, the main goal of this article has been to investigate whether the original results can be achieved by utilizing a different set of computational streams on the same data.

## 5 Conclusion

The aim of our study was to conceptually replicate the rsFC findings of an original study of early ocular blindness (Bauer et al., 2017). We were not able to fully replicate the results in the original study with our two proposed preprocessing pipelines, partly due to addition of more rigorous steps for removal of the noise and artifacts, and also likely due to different implementations of the same steps. Importantly, we highlighted some specific functional connection pairs in the study group that tend to arise regardless of the computational stream.

## Data Availability

The raw data supporting the conclusions of this article will be made available by the authors, without undue reservation.
