# Supplementary material for: Replicability of a resting-state functional connectivity study in profound early blindness
Source: Front Syst Neurosci. 2025 Apr 28;19:1547276. doi: 10.3389/fnsys.2025.1547276 (PMC12066490; doi:10.3389/fnsys.2025.1547276)
Supplement: Supplementary file 1 [file Data_Sheet_1.DOCX]

Supplementary Material

# Acronyms Associated with Regions of Interest

# A description of the acronyms used for the cortical regions of interest is shown in the table below.

Supplementary Table 1. Description of ROI Name Abbreviations.

| **ROI Name Abbreviation** | **ROI Full Name** | **ROI Name Abbreviation** | **ROI Full Name** |
| --- | --- | --- | --- |
| FrontPole | Frontal Pole | InfTemp | Inferior Temporal |
| MedOrbFront | Medial Orbitofrontal | MidTemp | Middle Temporal |
| LatOrbFront | Lateral Obitofrontal | SupTemp | Superior Temporal |
| ParsOrb | Pars Orbitalis | BanksSts | Banks of Superior Temporal Sulcus |
| ParsTrian | Pars Triangularis | TransTemp | Transverse Temporal |
| ParsOper | Pars Opercularis | Fusi | Fusiform |
| RostMidFront | Rostral Middle Frontal | Ento | Entorhinal |
| CaudMidFront | Caudal Middle Frontal | ParaHipp | Parahippocampal |
| SupFront | Superior Frontal | PostCent | Postcentral |
| ParaCent | Paracentral | SupraMarg | Supramarginal |
| PreCent | Precentral | SupPar | Superior Parietal |
| IsthCing | Isthmus Cingulate | InfPar | Inferior Parietal |
| PostCing | Posterior Cingulate | PreCuneus | Precuneus |
| CaudAntCing | Caudal Anterior Cingulate | LatOccip | Lateral Occipital |
| RostAntCing | Rostral Anterior Cingulate | Lingual | Lingual |
| Insula | Insula | PeriCalc | Pericalcarine |
| TempPole | Temporal Pole | Cuneus | Cuneus |

# Effect of Some Alternative Pipelines

For exploration, the between-group contrast was also evaluated by utilizing a subset of the preprocessing steps from RExt model, as indicated below.


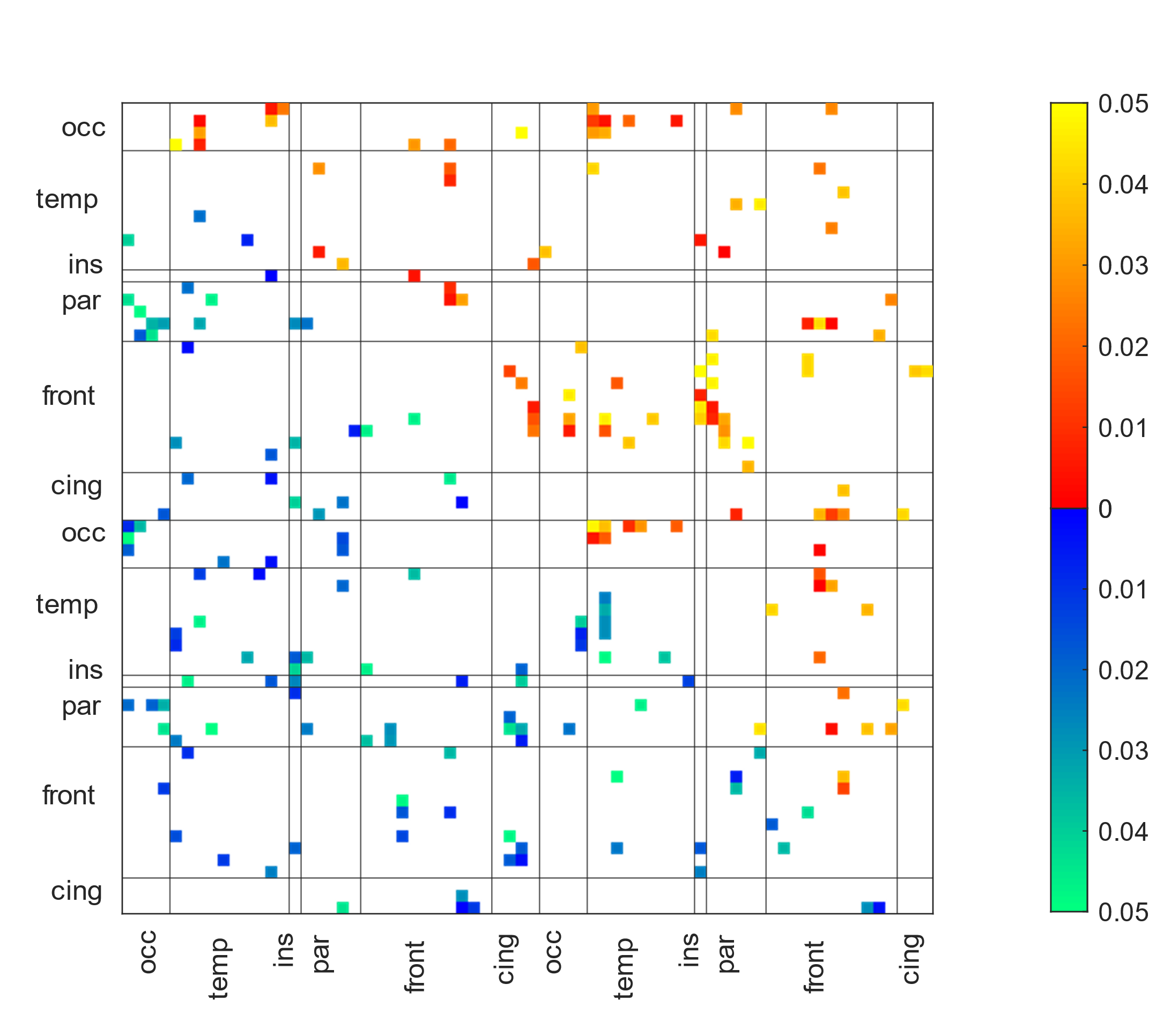


Supplementary Figure 1. The *p*-values associated with the between-group resting-state functional connectivity for EB > SC (warm colors) and EB < SC (cold colors), while removing the slice timing correction step from RExt preprocessing pipeline.


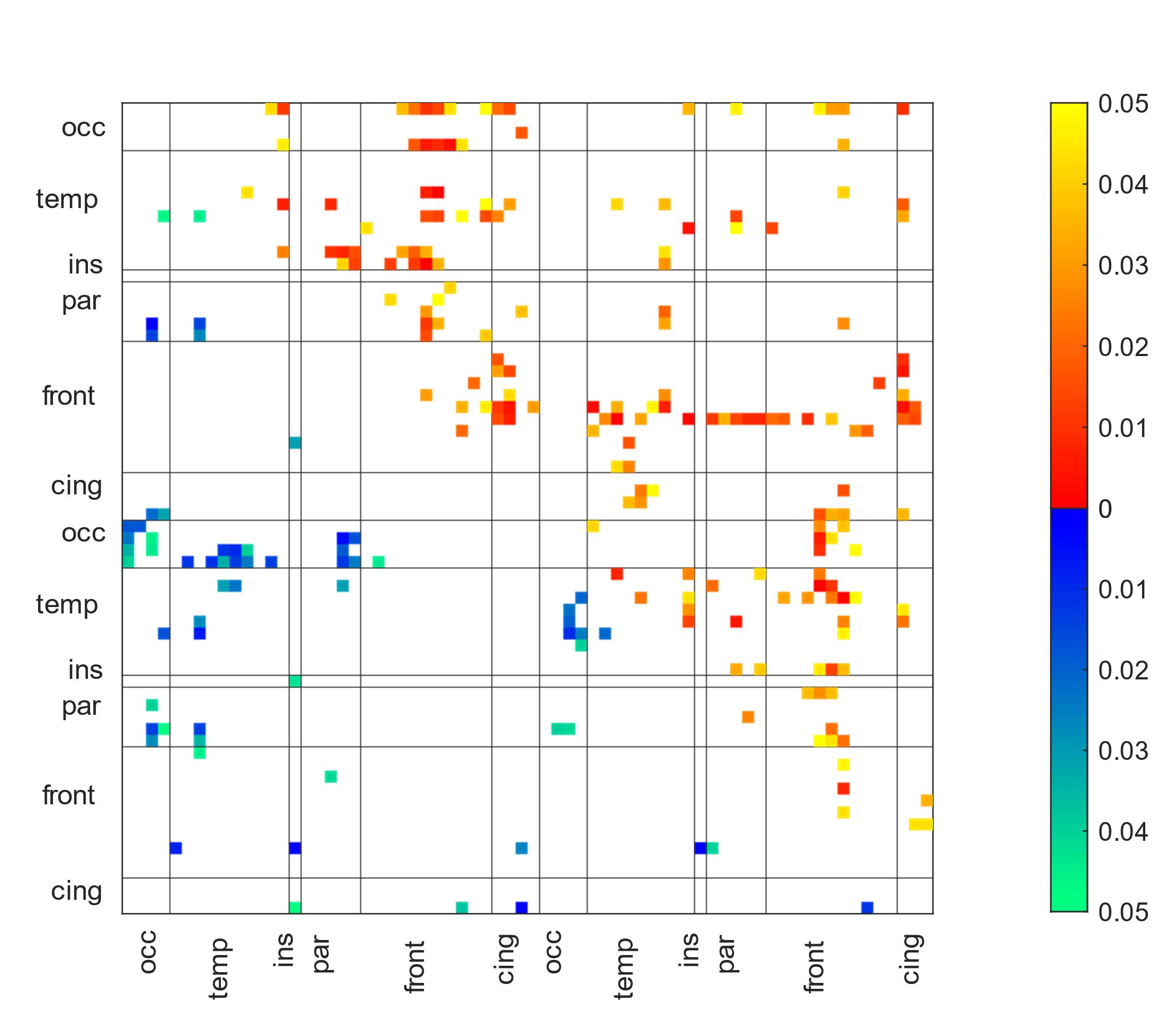


Supplementary Figure 2. The *p*-values associated with the between-group resting-state functional connectivity analysis are shown for EB > SC (warm colors) and EB < SC (cold colors), while removing the acompcor, i.e. anatomical component-based noise correction, step from RExt preprocessing pipeline.


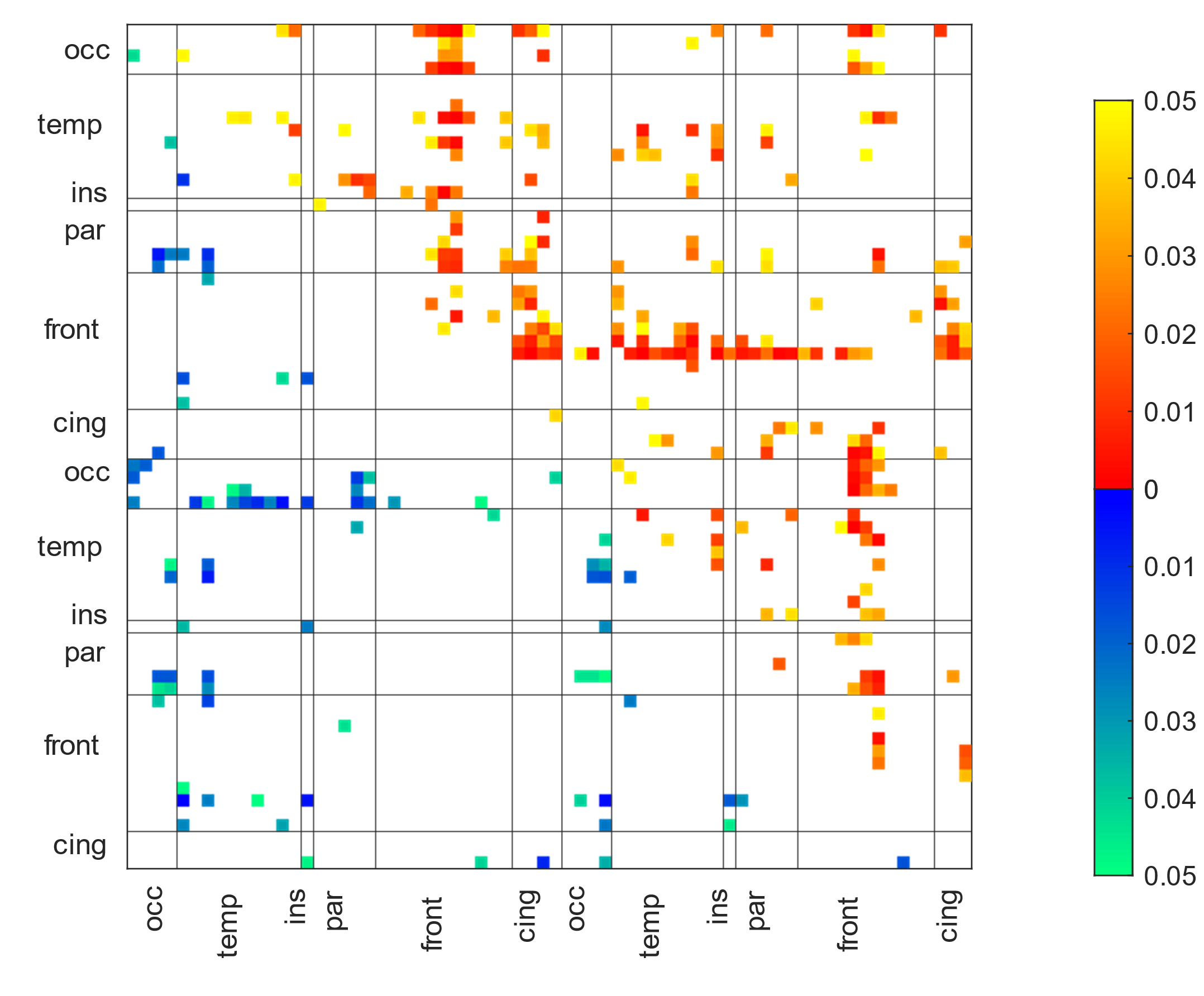


Supplementary Figure 3. The p-values associated with the between-group resting-state functional connectivity for EB > SC (warm colors) and EB < SC (cold colors), while removing the outlier scrubbing and acompcor, i.e. anatomical component-based noise correction, steps from RExt preprocessing pipeline.

# Between-group Top 1% Results

Connectivity pairs comprising the top 1% of the t-statistic values for the between-group contrasts were extracted for each pipeline and each group. Overlap was only observed between the original analysis and RMin model in the EB > SC contrast and only for four connectivity pairs as detailed in Supplementary Table 2 below.

Supplementary Table 2. Pairs accounting for the top 1% of t-statistic values observed in both the original and RMin model for EB > SC contrast.

| **Connectivity Pair** | **Source Lobe** | **Source ROI** | **Target Lobe** | **Target ROI** |
| --- | --- | --- | --- | --- |
| 1 | L Frontal | parsorbitalis | R Temporal | transversetemporal |
| 2 | L Frontal | parsorbitalis | R Parietal | postcentral |
| 3 | L Frontal | parsorbitalis | R Frontal | precentral |
| 4 | L Frontal | parsorbitalis | R Frontal | rostralmiddlefrontal |
